# HuR drives lung fibroblast differentiation but not metabolic reprogramming in response to TGF-β and hypoxia

**DOI:** 10.1186/s12931-021-01916-4

**Published:** 2021-12-28

**Authors:** Joshua Trivlidis, Noof Aloufi, Fatmah Al-Habeeb, Parameswaran Nair, Ilan Azuelos, David H. Eidelman, Carolyn J. Baglole

**Affiliations:** 1 Research Institute of the McGill University Health Centre, 1001 Decarie Blvd (EM22248), Montreal, QC H4A3J1 Canada; 2 Department of Pathology, McGill University, Montreal, QC Canada; 3 Department of Medicine, McGill University, Montreal, QC Canada; 4 Department of Pharmacology & Therapeutics, McGill University, Montreal, QC Canada; 5 Department of Medicine, McMaster University & St Joseph’s Healthcare, Hamilton, ON Canada

**Keywords:** Pulmonary fibrosis, Human antigen R, ELAVL1, Fibroblast, Hypoxia, TGF-β, Myofibroblast, Glycolysis

## Abstract

**Background:**

Pulmonary fibrosis is thought to be driven by recurrent alveolar epithelial injury which leads to the differentiation of fibroblasts into α-smooth muscle actin (α-SMA)-expressing myofibroblasts and subsequent deposition of extracellular matrix (ECM). Transforming growth factor beta-1 (TGF-β1) plays a key role in fibroblast differentiation, which we have recently shown involves human antigen R (HuR). HuR is an RNA binding protein that also increases the translation of hypoxia inducible factor (HIF-1α) mRNA, a transcription factor critical for inducing a metabolic shift from oxidative phosphorylation towards glycolysis. This metabolic shift may cause fibroblast differentiation. We hypothesized that under hypoxic conditions, HuR controls myofibroblast differentiation and glycolytic reprogramming in human lung fibroblasts (HLFs).

**Methods:**

Primary HLFs were cultured in the presence (or absence) of TGF-β1 (5 ng/ml) under hypoxic (1% O_2_) or normoxic (21% O_2_) conditions. Evaluation included mRNA and protein expression of glycolytic and myofibroblast/ECM markers by qRT-PCR and western blot. Metabolic profiling was done by proton nuclear magnetic resonance (^1^H- NMR). Separate experiments were conducted to evaluate the effect of HuR on metabolic reprogramming using siRNA-mediated knock-down.

**Results:**

Hypoxia alone had no significant effect on fibroblast differentiation or metabolic reprogramming. While hypoxia- together with TGFβ1- increased mRNA levels of differentiation and glycolysis genes, such as *ACTA2, LDHA,* and *HK2*, protein levels of α-SMA and collagen 1 were significantly reduced. Hypoxia induced cytoplasmic translocation of HuR. Knockdown of HuR reduced features of fibroblast differentiation in response to TGF-β1 with and without hypoxia, including α-SMA and the ECM marker collagen I, but had no effect on lactate secretion.

**Conclusions:**

Hypoxia reduced myofibroblasts differentiation and lactate secretion in conjunction with TGF-β. HuR is an important protein in the regulation of myofibroblast differentiation but does not control glycolysis in HLFs in response to hypoxia. More research is needed to understand the functional implications of HuR in IPF pathogenesis.

## Background

Interstitial lung diseases (ILDs) are an array of lung disorders that involve varying degrees of inflammation and fibrosis of the lung parenchyma [1, 2]. The most fatal form of ILD is idiopathic pulmonary fibrosis (IPF), a chronic, progressive disease distinguished by abnormal accumulation of fibrotic tissue [2, 3]. Although the cause of IPF is not known, various environmental, microbial, and genetic factors are proposed to play important roles in IPF pathobiology. The current paradigm of pathogenesis for IPF is that recurrent damage to the alveolar epithelium promotes an abnormal wound-healing response that results in fibrosis rather than repair [4]. It is thought that damage to the alveolar epithelium drives the accumulation of myofibroblasts, which are α-smooth muscle actin (α-SMA)-expressing cells that produces copious amounts of extracellular matrix (ECM) proteins such as collagens (COL) and fibronectin (FN), which contribute to dysfunctional tissue remodeling [5].

The differentiation of fibroblasts into ECM-producing myofibroblasts occurs under the direction of cytokines, particularly transforming growth factor-β (TGF-β). TGF-β is produced as a latent protein that requires activation by factors such as mechanical stretch and changes in pH [6]. This change in extracellular pH can be due to a switch from oxidative phosphorylation to aerobic glycolysis, a state in which predominant uptake/use of glucose for energy persists despite the presence of adequate oxygen for mitochondrial respiration [7, 8]. The conversion of glucose to lactate contributes to an acidic extracellular environment as the lactate is excreted from the cell [9]. There is emerging evidence that the development of aerobic glycolysis in IPF occurs when the acidic microenvironment promotes myofibroblast differentiation, in part, through pH-dependent activation of latent TGF-β [10] 11. Furthermore, excessive accumulation of myofibroblasts and ECM within the alveolar space creates hypoxic conditions.

In both instances, glycolysis can be driven by hypoxia inducible factor-1α (HIF-1α), an oxygen-sensitive transcription factor. HIF-1α increases some of the key glycolytic genes including glycolytic enzymes hexokinase-2 (HK2/HKII) and lactate dehydrogenase A (LHDA/ LDH5) [12–14]; LDHA converts pyruvate to lactate during glycolysis. Mechanistically, HIF-1 is classically known to be activated under hypoxic conditions where it is protected from proteolytic degradation. HIF-1α can also be regulated by human antigen R (HuR) [15–17], a member of the Hu/embryonic lethal, abnormal vision (ELAV) family of RNA-binding proteins [18]. HuR is best-known to stabilize target mRNA by binding to mRNA with an adenylate-and uridylate- rich element (ARE) in the 3’ untranslated region (3’UTR) [19]. Binding of HuR to the 3’UTR of target mRNA facilitates nuclear export and prevents degradation of the mRNA, which increases cellular protein levels by allowing the message to be efficiently translated. We have recently shown that HuR controls myofibroblast differentiation and ECM production in response to TGF-β in primary human lung fibroblasts (HLFs) [20]. We now question whether under hypoxic conditions, HuR plays a role in controlling metabolic reprogramming in addition to TGF-β-induced myofibroblast differentiation. Herein, we describe the unexpected findings that hypoxia reduces TGF-β-induced myofibroblast differentiation and glycolytic reprogramming, but that the switch to glycolysis occurred independently of HuR. This study is the first to investigate the role of HuR in myofibroblast differentiation and metabolic reprogramming in response to hypoxia, which could help provide the basis for new targeted therapy in fibrotic disease such as IPF.

## Methods

### Derivation and culture of HLFs

Primary HLFs were derived from lung tissue obtained through lung resection surgery in subjects undergoing the procedure at McMaster University as previously described [21]. This study was conducted on HLFs from three different subjects with no smoking history and or other known risk factors (e.g., radiation therapy) for lung fibrosis. Cells were cultured in MEM (Thermo Fisher Scientific, USA) containing 10% fetal bovine serum (FBS; Hyclone Laboratories, Logan, UT) supplemented with gentamycin (WISENT Inc, Canada) and Antibiotics-Antimycotics (WISENT Inc, Canada). HLFs were used with a cell passage number between 6 and 9.

### Cell treatments

To mimic normoxia, HLFs were incubated in humidified chambers at 37 °C and 21% O_2_ /5% CO_2_. Under hypoxic conditions, HLFs were incubated at 37 °C and 1% O_2_ /5% CO_2_ using the Xvivo System Model X3 hypoxia incubator (BioSpherix Ltd, USA). Under both normoxia and hypoxia conditions, HLFs were either left untreated or treated with human recombinant TGF-β1 (R&D Systems, USA). Incubation times are indicted for each experimental outcome.

### Immunofluorescence (IF)

HLFs were treated under normoxia, hypoxia, TGF-β or hypoxia plus TGF-β1 for 4 h, followed by fixation with paraformaldehyde for 15 min and permeabilization for 30 min in PBS containing 0.5% Triton. Once HLFs were incubated with blocking buffer (Dako, USA) for 1 h at room temperature, cells were incubated in a 1:300 dilution of anti-HuR antibody (Santa Cruz, USA) in antibody diluent (Dako, USA) or with antibody diluent only for 2 h at room temperature. After PBS washes, cells were incubated in a 1:1000 dilution of secondary antibody Alexa fluor 555 (Invitrogen, USA). Then, HLFs were washed with PBS and nuclei were stained in a 1:1000 dilution of Hoechst 33342 (Thermo Fisher, USA). Images were acquired through the Zeiss LSM 780 confocal microscope (Zeiss, Germany). ICY software was used for bioimage analysis to quantify HuR cytoplasmic translocation.

### Quantitative RT-PCR (qPCR)

HLFs were cultured with serum free MEM for 18 h prior to treatment under normoxia, hypoxia, TGF-β1 or hypoxia plus TGF-β1 for 24 and 48 h. Then, total RNA was isolated with Trizol. Quantification of RNA was done by using a Nanodrop 1000 spectrophotometer infinite M200 pro (Tecan, Switzerland). Reverse transcription was accomplished using iScript Reverse Transcription Supermix (Bio-Rad laboratories, Canada) and mRNA levels of *ELAVL1, ACTA2, FN1, COL1A1, LDHA* and *HKII* were analyzed using gene specific primers (Table 1). qPCR was carried out by combining 1 μl cDNA and 0.5 μM primers with SsoFast EvaGreen (Bio-Rad Laboratories, Canada) and amplification was performed using a CFX96 Real-Time PCR Detection System (Bio-Rad Laboratories). Thermal cycling was initiated at 95 °C for 3 min and was followed by 39 cycles of denaturation at 95 °C for 10 s and annealing at 59 °C for 5 s. Genomic RNA expression was analyzed using the ΔΔCt method, and results are presented as fold-change normalized to the housekeeping gene (*S9*).

**Table 1:** Primer sequences used for qRT-PCR analysis

Gene	Forward Primer Sequence	Reverse Primer Sequence
*ELAVL1*	AACGCCTCCTCCGGCTGGTGC	GCGGTAGCCGTTCAGGCTGGC
*COL1A1*	CAGACTGGCAACCTCAAGAA	CAGTGACGCTGTAGGTGAAG
*ACTA2*	GACCGAATGCAGAAGGAGAT	CACCGATCCAGACAGAGTATTT
*FN1*	CTGAGACCACCATCACCATTAG	GATGGTTCTCTGGATTGGAGTC
*S9*	CAGCTTCATCTTGCCCTCA	CTGCTGACGCTTGATGAGAA
*LDHA*	GGAGATTCCAGTGTGCCTGT	CGTAAAGACCCTCTCAACCACC
*HKII*	GGGCGGATGTGTATCAAT	GTGAGCCCATGTCAATCT

### Western blot

Total cellular protein was extracted using RIPA lysis buffer (Thermo Fisher Scientific, USA) in conjunction with Protease Inhibitor Cocktail (Roche, USA). Protein lysates were electrophoresed on 10% SDS-PAGE gels and transferred onto Immuno-blot PVDF membranes (Bio-Rad Laboratories, USA). After transfer, the membrane was blocked with a blocking solution of 5% w/v non-fat dry milk in 1 × PBS/0.1% Tween-20 for one hour at room temperature. Antibodies were applied to membranes for 1 h or overnight. The following antibodies used were: anti-HuR (1:2000; Santa Cruz, USA), anti-α-SMA (1:5000; Sigma-Aldrich, USA), anti-Col1A1 (1:200; Santa Cruz, USA), anti-HIF-1α (1:1000; Abcam, USA), anti-FN (1:200; Santa Cruz, USA), anti-HKII (1:1000; Cell Signaling), anti-LDHA (1:1000; Cell Signaling) and anti-Tubulin (1:50,000; Sigma-Aldrich, USA). Secondary antibodies were anti-rabbit IgG, horseradish peroxidase (HRP) linked (1:10,000; Cell Signaling Technologies, USA) and HRP conjugated anti-mouse IgG (1:10,000; Cell Signaling Technologies, USA). Membrane visualization was performed by using Clarity western enhanced chemiluminescence (ECL) substrate (Bio-Rad Laboratories, Canada) or SuperSignal West Femto Maximum Sensitivity Substrate (Thermo Scientific, USA). Detection of protein bands was done by the ChemiDoc MP Imaging System (Bio-Rad Laboratories). Densitometric analysis was done using Image Lab Software Version 5 (Bio-Rad Laboratories) and protein expression was normalized to tubulin and presented as fold change compared to normoxia.

### Cytoplasmic and nuclear protein fractionation

Cytoplasmic and nuclear protein fractions were obtained using a nuclear extraction kit as per manufacturer instructions (Active Motif, Carlsbad, CA). Protein concentrations were determined by the BCA protein assay kit. Western blot and antibodies used were described above. Lamin A/C (1:1000; Cell Signaling Technologies, CA) was used as a marker for the nuclear fraction and β-Tubulin (1:50,000; Sigma, CA) was used as a marker for the cytoplasmic fraction.

### Proton nuclear magnetic resonance (^1^H NMR)

Cell supernatants were collected and supplemented with 10% D_2_O for shift lock and 1 mM total soluble protein (TSP) standard (Sigma Aldrich). The ^1^H spectra were obtained using a 500-MHz Bruker machine using a pre-saturation method following 128 scans. Spectra analysis was performed using ACD Labs software (Advanced Chemistry Development UK Ltd, UK) and integrals for peaks at 5.2 ppm (glucose) and 1.3 ppm (lactate) were quantified in relation to the standard internal TSP peak at 0.0 ppm.

### RNA immunoprecipitation-qPCR (RIP-qPCR)

HLFs were grown to approximately 70–80% confluence and cultured with serum-free MEM for 18 h before the treatment after which cells were rinsed with PBS and collected by cell scraper in PBS. Cells were centrifuged at 1500 rpm, 4 °C for 5 min, then the PBS discarded. Cell pellets were harvested in the lysis buffer as previously described [20], incubated for 15 min on ice and then centrifuged at 10,000 rpm, 4 °C for 15 min. The cell extracts were transferred into a new tube and the protein concentration was measured. Thirty-five μl of protein G Sepharose™ 4 fast glow beads (GE Healthcare) were pre-coated with 2 μg of IgG (Cell Signaling Technologies, CA) or 2 μg of anti-HuR (Santa Cruz Biotechnology) antibodies overnight on a rotator at 4 °C. Beads were washed, incubated with cell extracts for 2 h at 4 °C and washed again to remove unbound material. RNA was then extracted, reverse transcribed and analyzed by qRT-PCR as described above. RNA expression was normalized to *S9* mRNA bound in a non-specific manner to IgG [22, 23].

### HuR-siRNA knockdown

HLFs were seeded at 10 × 10^4^ cells/cm^2^ and one day later were transfected with 60 nM of small-interfering RNA (siRNA) targeting HuR (Santa Cruz, USA) or non-targeting control siRNA (Santa Cruz) using Lipofectamine RNAiMAX Transfection Reagent (Thermo Fisher Scientific, USA). After one hour, 10% MEM medium was added onto the cells. Twenty-four hours later, fibroblasts were treated with serum-free MEM medium for 18 h, followed by treatment under normoxia, hypoxia, TGF-β1 or hypoxia plus TGF-β1 for 72 h.

### Statistical analysis

Statistical analysis was performed using one or two-way analysis of variance (ANOVA) with Bonferonni's or Tukey’s multiple comparisons using GraphPad Prism 6 (v.602; GraphPad Software Inc, USA). Statistical significance was considered in all cases which had a p value < 0.05.

## Results

### Hypoxia attenuates TGFβ1-induced metabolic shift towards glycolysis

**Fig. 1:**
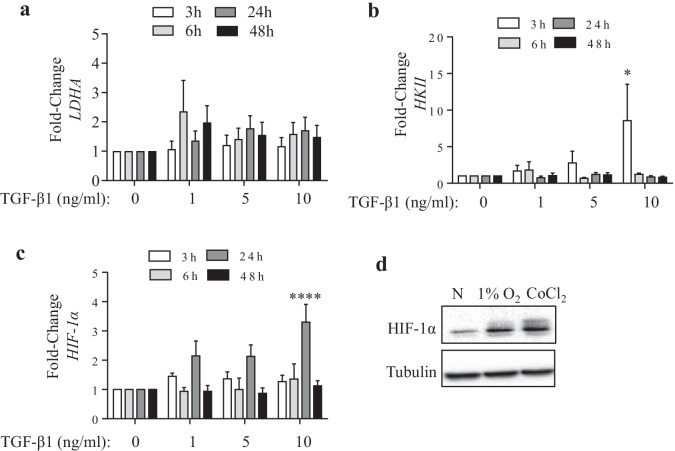
TGF-β1 dose-dependently increases mRNA expression of genes involved in metabolic reprogramming. Fold change was calculated utilizing the ΔΔCt method normalized to *GAPDH* for *LDHA* (**a**), *HKII* (**b**) and *HIF-1α* (**c**). There was a significant induction in *HKII* and *HIF-1α* only at the highest concentration of TGF-β used (10 ng/ml). Each sample is normalized to the untreated control for that same time-point (*p value ˂ 0.05; ****p ˂ 0.0001). Results are expressed as the mean ± SEM, n = 6 independent experiments. **d** There was an increase in protein levels of HIF-1α in response to hypoxia and CoCl_2_ for 6 h. Representative western blot is shown

To first establish the extent to which TGF-β causes changes in the expression of genes involved in metabolic reprogramming, HLFs were treated with TGF-β1 at varying concentrations for up to 48 h prior to analysis of gene expression. We chose to study the expression of *LDHA, HKII* and *HIF-1α* as these are important regulatory enzymes in the glycolytic pathway. TGF-β1 caused no statistically significant change in the mRNA expression of LDHA (Fig. 1a) but significantly increased the expression of *HKII* mRNA only at the highest concentration (10 ng/ml) (Fig. 1b). *HIF-1α* mRNA expression was also significantly induced by TGF-β1 at this concentration (Fig. 1c). To ensure that the experimental conditions to be utilized herein were in fact causing a hypoxic response, we also performed western blots for HIF-1α protein after exposure to hypoxia or CoCl_2_. CoCl_2_ not only acts as iron chelator and replaces iron by cobalt at the iron-binding center of prolyl hydroxylase to reduce the hydroxylation activity, but also blocks the von Hippel-Lindau tumor suppressor protein (pVHL) binding to the PAS domain of HIF-1α; this allows HIF-1α to escape degradation by a ubiquitin-proteasomal pathway and hence increases its protein levels [24, 25]. Exposure to hypoxia as well as CoCl_2_ for 6 h increased HIF-1α protein (Fig. 1d). Because our goal was to assess the impact of combined exposure of hypoxia plus TGF-β1 on lung fibroblasts, we utilized TGF-β1 at a concentration of 5 ng/ml for the remainder of the experiments, as this concentration did not significantly change the mRNA expression of these metabolic genes.

**Fig. 2:**
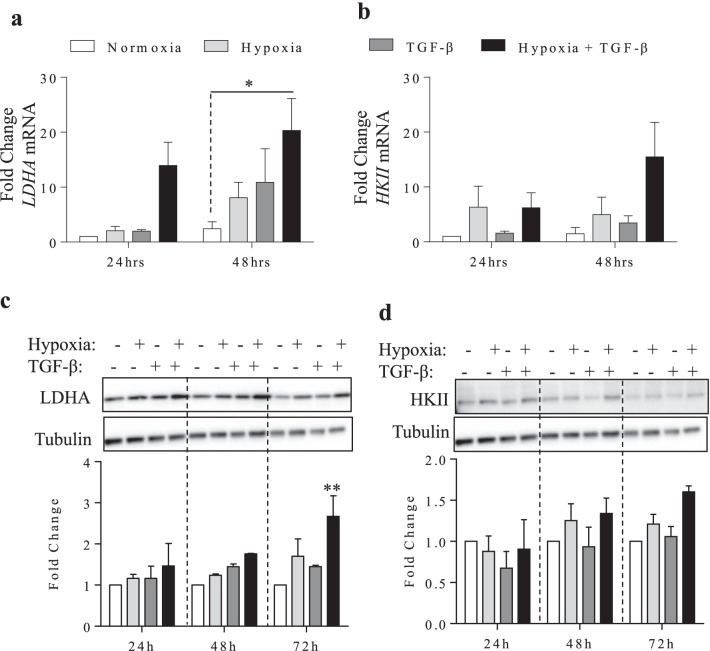
Alterations in components of the glycolytic pathway. **a** There was a significant increase in *LDHA* mRNA levels following treatment with hypoxia with TGF-β1. **b** There was a modest increase in *HKII* mRNA after treatment with hypoxia and TGF-β1. Values are represented as the mean ± SEM (n = 3–4 independent experiments utilizing cells from 3 separate subjects). Means are expressed as fold change from the control (24 h- normoxia; *p < 0.05 compared to control). **c** There was a significant increase in LDHA protein in response to TGF-β together with hypoxia compared to normoxia. **d** HKII protein did not significantly changes with exposures

We next focused on the effects of hypoxia together with TGF-β1, and first analyzed the mRNA expression of *LDHA* and *HKII* in HLFs exposed to normoxia (21%), hypoxia (1%), TGF-β1 (5 ng/ml) as well as hypoxia together with TGF-β1. At the mRNA level, there was a significant increase in *LDHA* mRNA after treatment with hypoxia in conjunction with TGF-β1 at 48 h (Fig. 2a). Although there was an elevated tendency in *HKII* expression under conditions of hypoxia and TGF-β1, this did not reach statistical significance (Fig. 2b). As in Fig. 1, there was a no significant induction in *LDHA* or *HKII* mRNA in response to TGF-β alone. At the protein level, LDHA (Fig. 2c) and HKII (Fig. 2d) were constitutively expressed. There was only a significant increase in in LDHA in response to both hypoxia and TGF-β at 72 h; HKII remained unchanged with these exposures.

**Fig. 3:**
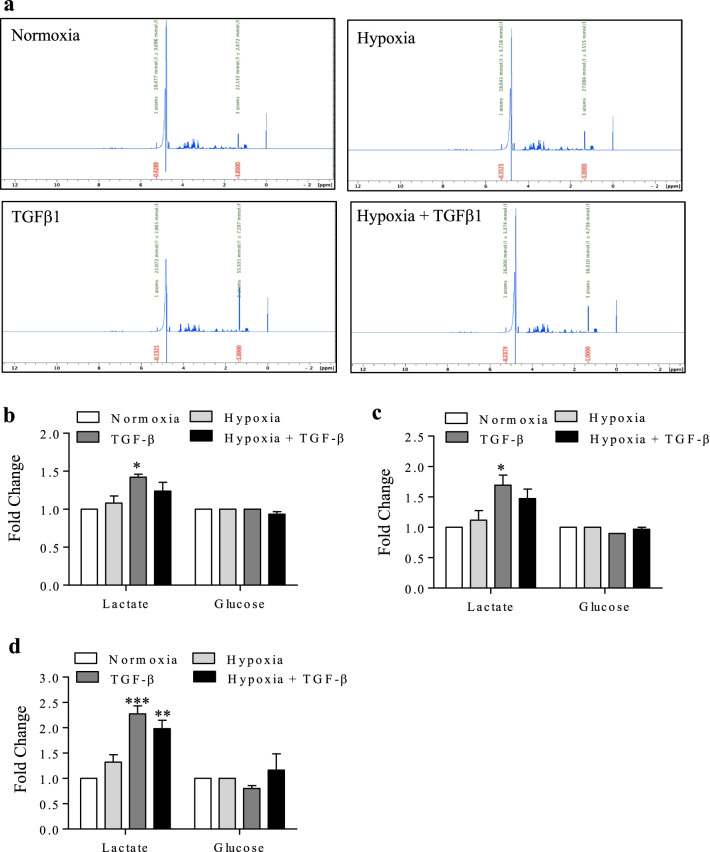
Hypoxia decreases lactate production in conjunction with TGF-β1. **a** Representative spectra from the 72 h time-point for normoxia, hypoxia, TGFβ1- and hypoxia with TGF-β. Lactate (1.3 PPM) and glucose (5.2 PPM) peaks were integrated in comparison to benzoic acid to determine the concentration (mmol/l), labelled in green, of both molecules in every sample by ^1^H NMR spectroscopy. **b** Quantification of the fold change in lactate and glucose concentrations at 24 h. **c** Quantification of fold change in lactate and glucose concentrations at 48 h. **d** Quantification of fold change in lactate and glucose concentrations at 72 h. Values are represented as the mean ± SEM (n = 3 independent experiments using cells from 3 separate subjects); *p < 0.05, ** p < 0.01 and ***p < 0.001

We then utilized ^1^H NMR to analyze lactate production relative to glucose consumption. We measured two separate peaks, scaled in parts per million (ppm): glucose (5.2 ppm) and lactate (1.3 ppm) (Fig. 3a). To quantify the rate of lactate secretion relative to the amount of glucose present in the cell-conditioned media, we used a known concentration of benzoic acid to establish a standard, followed by the integration of both the glucose and lactate peaks to calculate their individual concentrations. The integrated concentration (mmol/l) is labeled in green above both peaks (Fig. 3a). When reading from left to right, the first peak denotes the concentration of glucose while the following peak represents lactate concentration. We calculated the difference in glycolysis corresponding to each treatment at 24, 48 and 72 h. We show ^1^H NMR spectra from the 72 h time point (Fig. 3a), as this period reflected the largest changes in lactate secretion. We detected a significant increase in lactate production at 24- (Fig. 3b), 48 (Fig. 3c)- and 72- (Fig. 3d) hours following treatment with TGF-β1. We did not find any significant changes with hypoxia alone, although we noted a slight trend in increased lactate compared with normoxia. There was no significant change in lactate production in response to TGF-β plus hypoxia at the 24 and 48 h timepoints although there was an increase by 72 h. Thus, our results show that there is a significant shift in metabolism towards glycolysis caused by TGF-β1 alone that is blunted under hypoxic conditions despite upregulation of key enzymes involved in metabolic reprogramming.

### TGF-β1-induced fibroblast differentiation to myofibroblasts is reduced in response to hypoxia

**Fig. 4:**
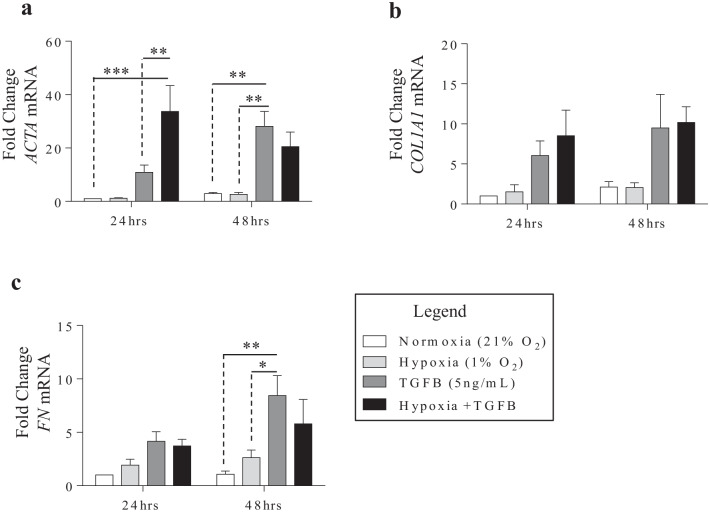
Increased mRNA of *ACTA2*, *COL1A1* and *FN1* in response to TGF-β1 and hypoxia with TGF-β1. **a** *ACTA2* mRNA signficantly increased by TGF-β1 at 48 h and by hypoxia with TGF-β1 at 24- and 48 h. The effect of TGF-β1 combined with hypoxia is significantly greater than TGF-β1 alone at 24- but not 48 h. **b** The expression of *COL1A1* mRNA do not significantly change, although an increasing trend was observed in response to TGF-β1 alone and when combined with hypoxia. **c** TGF-β1 increased *FN* mRNA levels significantly at 48 h compared to normoxia and hypoxia. Values are represented as the mean ± SEM (n = 4 independent experiments utilizing cells from 3 separate subjects); *p < 0.05, **p < 0.01 and ***p < 0.001

Our next question focused on the effect of hypoxia on the differentiation of fibroblasts to myofibroblast in response to TGF-β, a process believed to be involved in IPF pathogenesis [26]. To address whether hypoxia contributes to myofibroblast differentiation, we compared TGF-β1 with and without exposure to hypoxia and assessed the differentiation of HLFs by analyzing the expression of α-SMA (*ACTA2*), a marker of myofibroblasts, as well as key ECM components including collagen 1 (*COL1A1*) and fibronectin 1 (*FN*) by qPCR and western blot. At the mRNA level, TGF-β1 increased *ACTA2* and *FN* by 48 h (Fig. 4a and c). Therefore, we conclude that hypoxia, either alone or in combination with TGF-β, has limited impact on the induction of genes associated with myofibroblast differentiation and ECM production.

**Fig. 5:**
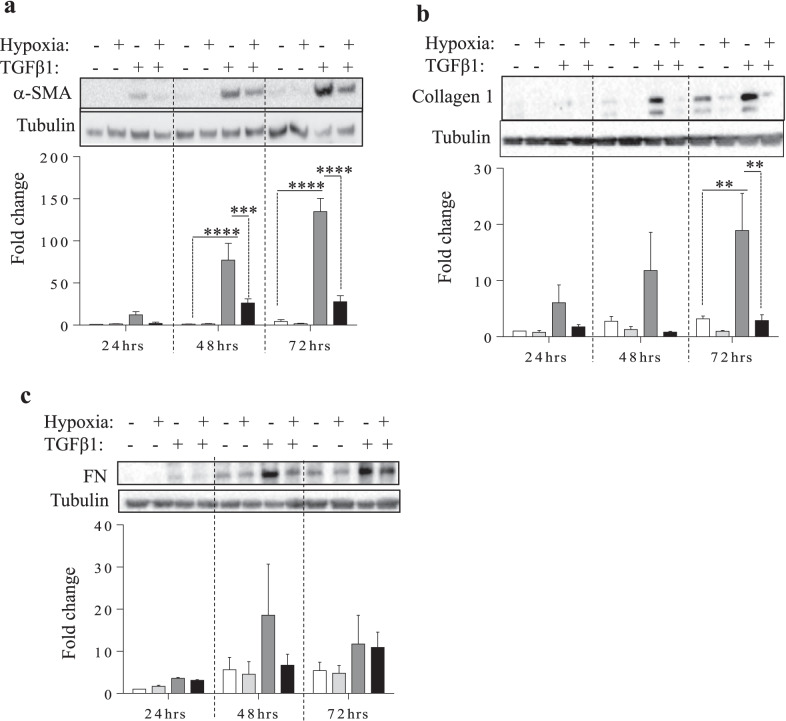
TGF-β1-induced increase α-SMA and Collagen 1 protein is attenuated by co-exposure to hypoxia. Upregulation of α-SMA **a** as well as collagen 1 **b** occurred in a time dependant manner in response to TGF-β1. This increase is attenuated following inclusion of hypoxia. There is no significant change in fibronectin **c** protein levels in response to hypoxia, TGF-β1 or hypoxia with TGF-β1. Values are represented as the mean ± SEM (n = 3–4 independent experiments using cells from 3 separate subjects); **p < 0.01; ***p < 0.001 and ****p < 0.0001

At the protein level, TGF-β1 significantly increased α-SMA at 48- and 72-h, with a noticeable induction at 24 h (Fig. 5a). A similar trend occurred with Collagen I, where there was a significant increase at 72 h (Fig. 5b). The protein level of FN did not change significantly at all time points examined (Fig. 5c). Hypoxia significantly reduced the TGF-β1-induced expression of α-SMA (Fig. 5a) and Collagen I protein (Fig. 5b). There was no effect noted for FN (Fig. 5c). Overall, these results show that TGF-β1-induced glycolytic flux and fibroblast differentiation are attenuated in hypoxic conditions.

### HuR translocation to the cytoplasm is increased by hypoxia

**Fig. 6:**
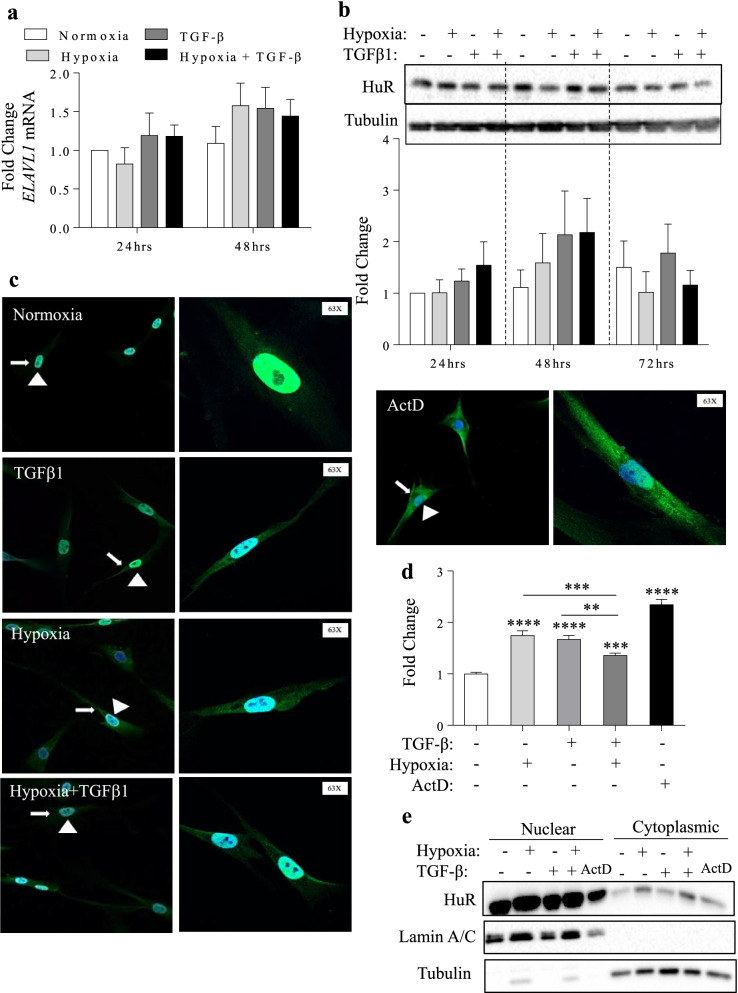
HuR expression and localization in response to hypoxia and TGF-β1. **a** mRNA levels and **b** protein levels of HuR in HLFs were assessed 24–72 h following treatment with hypoxia, TGF-β1 and hypoxia with TGF-β1. Values are represented as the mean ± SEM (n = 4–6 independent experiments from 3 separate subjects). **c** HuR localization using immunofluorescence at 20 × magnification (left panels) shows cytoplasmic translocation in HLFs treated with hypoxia, TGF-β1 and hypoxia with TGF-β1. White arrowheads designate blue nuclear staining (Hoescht) while white arrows indicate green cytoplasmic staining. Note the 63 × magnification images (right panels) showing representative cells and associated HuR localization. ActD was used as a positive control for HuR translocation to the cytoplasm. Images were pseudo-colored green for visualization purposes. **d** HuR quantification of IF. Values are represented as the mean ± SEM (n = 3–4 independent experiments from 3 separate subjects; **p˂0.01; ***p < 0.001, and ****p < 0.0001. **e** There was a noticeable increase in cytoplasmic levels of HuR with hypoxia with and without TGF-β. Representative western blot is shown

We have previously reported that HuR is essential for the differentiation of lung fibroblasts into myofibroblasts in response to TGF-β1 [20]. Therefore, we examined whether the observed effects of hypoxia in attenuating TGF-β-induced metabolic reprogramming and myofibroblast differentiation are also mediated by HuR. Neither hypoxia, TGF-β1, nor hypoxia with TGFβ1 significantly changed the total cellular expression of HuR at the mRNA (Fig. 6a) or protein levels (Fig. 6b). However, a major function of the HuR lies in its ability to stabilize mature target mRNA in the cytoplasm, thereby ensuring mRNA stability by providing protection from degradation machinery [17, 27]. The effect of hypoxia, with or without TGF-β1, on HuR localization is currently unknown. Therefore, we treated primary HLFs from three separate subjects with hypoxia, TGF-β1 or hypoxia with TGF-β1 for 4 h and utilized IF microscopy to assess the cellular localization of HuR. Figure 6c illustrates that under normoxic conditions, HuR is predominantly nuclear (*arrowheads*) while under hypoxic treatment, we detected higher cytoplasmic levels of HuR (Fig. 6c). Similarly, cytoplasmic HuR is elevated in fibroblasts treated with TGF-β1 alone. An increase in cytoplasmic HuR can also be seen in fibroblasts treated with hypoxia combined with TGF-β1 (Fig. 6c). As a positive control, Actinomycin D (ActD) induced almost complete translocation of HuR from the nucleus to the cytoplasm (Fig. 6c) [28]. Quantification revealed that there was a significant increase in cytoplasm localization of HuR under hypoxia, TGF-β1 as well as hypoxia with TGF-β1 compared to normoxia (Fig. 6d). There was less translocation of HuR to the cytoplasm in response to hypoxia with TGF-β1 compared to either hypoxia or TGF-β1 alone (Fig. 6d). Similar results were obtained by western blot of cytoplasmic extracts (Fig. 6e). In summary, HuR translocates to the cytoplasm in response to hypoxia, TGF-β1 and hypoxia with TGF-β1 in primary HLFs but the relative level of cytoplasmic localization was reduced by hypoxia.

### HuR is required for fibroblast differentiation and ECM production

**Fig. 7:**
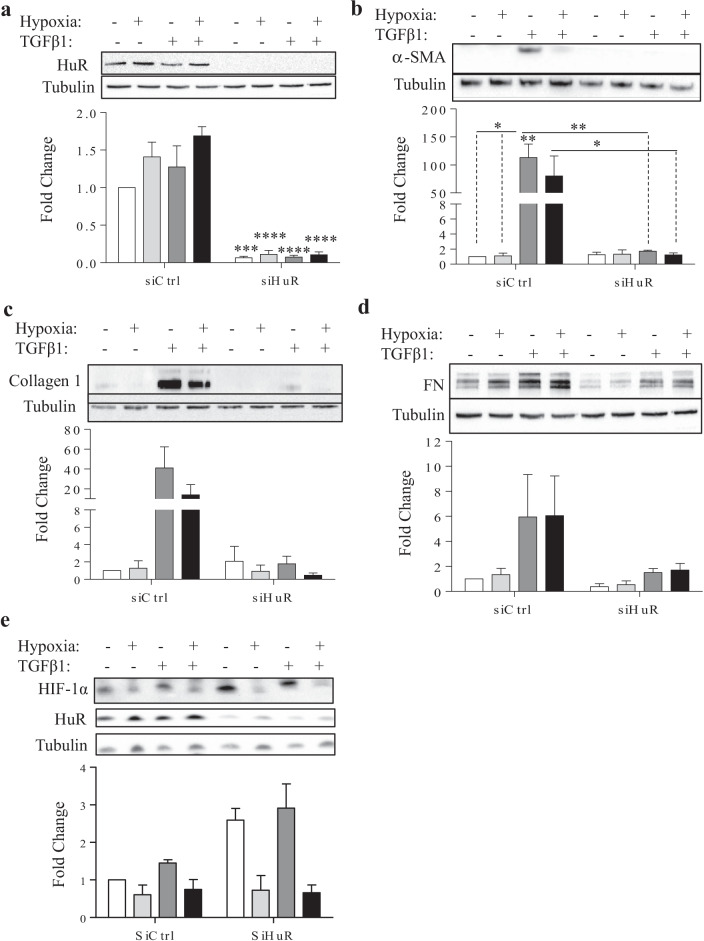
HuR knock-down attenuates TGF-β1-induced increase of α-SMA and Collagen I in HLFs. **a** HuR siRNA significantly decreased HuR protein levels. TGF-β1-induced increase in protein levels of α-SMA **b** and collagen I **c** were downregulated significantly following HuR knock-down. **d** There was a trend towards a decrease in FN protein level following HuR siRNA. **e** HIF-1α was not altered by exposure to hypoxia with or without TGF-β1 at this timepoint in siCtrl cells. There was more HIF-1α in siHuR cells except in response to hypoxia. Values are represented as the mean ± SEM (n = 2–4 independent experiments using cells from 3 separate subjects); *p < 0.05, **p < 0.01, ***p < 0.001 and ****p < 0.0001

Our next question revolved around the involvement of HuR in fibroblast differentiation and ECM production in lung fibroblasts. To test this, we reduced total HuR protein levels using siRNA by more than 75% (Fig. 7a). This decrease in HuR significantly attenuated the TGFβ1-induced increase in α-SMA and Collagen 1 protein levels (Fig. 7b and c), similar to our previous findings at the 72-h timepoint [20]. HuR knockdown also significantly decreased both α-SMA and collagen 1 protein levels in HLFs treated with hypoxia and TGF-β1. FN levels after TGF-B1, with or without hypoxia, appeared to be lower after knockdown of HuR but this did not reach statistical significance (Fig. 7d). HIF-1α levels were comparable between treatments at this timepoint in control cells (Fig. 7e); knockdown of HuR increased basal levels of HIF-1α but this increase did not occur in siHuR fibroblasts exposed to hypoxic conditions. These data indicate hypoxia does not alter the role of HuR in TGF-β-induced myofibroblast differentiation.

### Hypoxia does not affect HuR binding to target mRNA

**Fig. 8:**
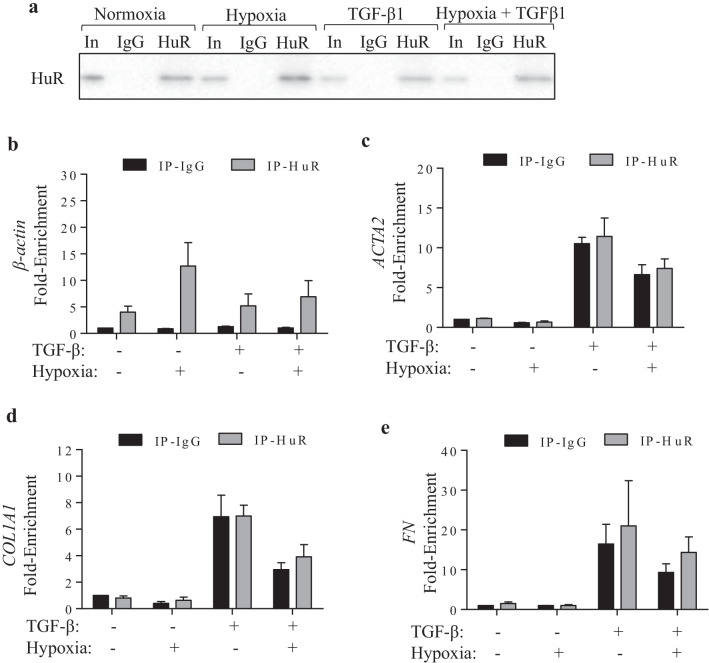
Hypoxia does not affect binding of HuR to select mRNA. **a** Representative western blot of HuR immunoprecipitation (IP). Input is whole cell lysate. IgG is the IP with the control antibody and HuR refers to IP with the anti-HuR antibody. Note that the enrichment of *β-actin* (**b**), *ACTA2* (**c**), *COL1A1* (**d**) and FN **(e)** is unaffected by exposure to hypoxia. Values are represented as the mean ± SEM (n = 4 independent experiments)

We previously published that HuR binds to mRNA involved in myofibroblast differentiation and ECM production [20]. To next evaluate if hypoxia affects the ability of HuR to bind to mRNA. we used RIP-qPCR [22, 23, 29], an antibody-based technique used to map RNA–protein interactions. Verification of HuR IP is in Fig. 8a. As a control, we evaluated HuR binding to a known target gene- *β-actin* (Fig. 8b) whose binding occurs under all conditions examined. Our data show that hypoxia, alone or in combination with TGF-β, had no affect on HuR binding to *β-actin* (Fig. 8b), *ACTA2* (Fig. 8c), *COL1A1* (Fig. 8d) and *FN* (Fig. 8e) mRNA. These data suggest that alterations in the ability of HuR to bind to its target mRNA cannot account for differences between mRNA and protein expression associated with hypoxic exposure.

### HuR does not play a significant role in a TGF-β1-induced metabolic shift

**Fig. 9:**
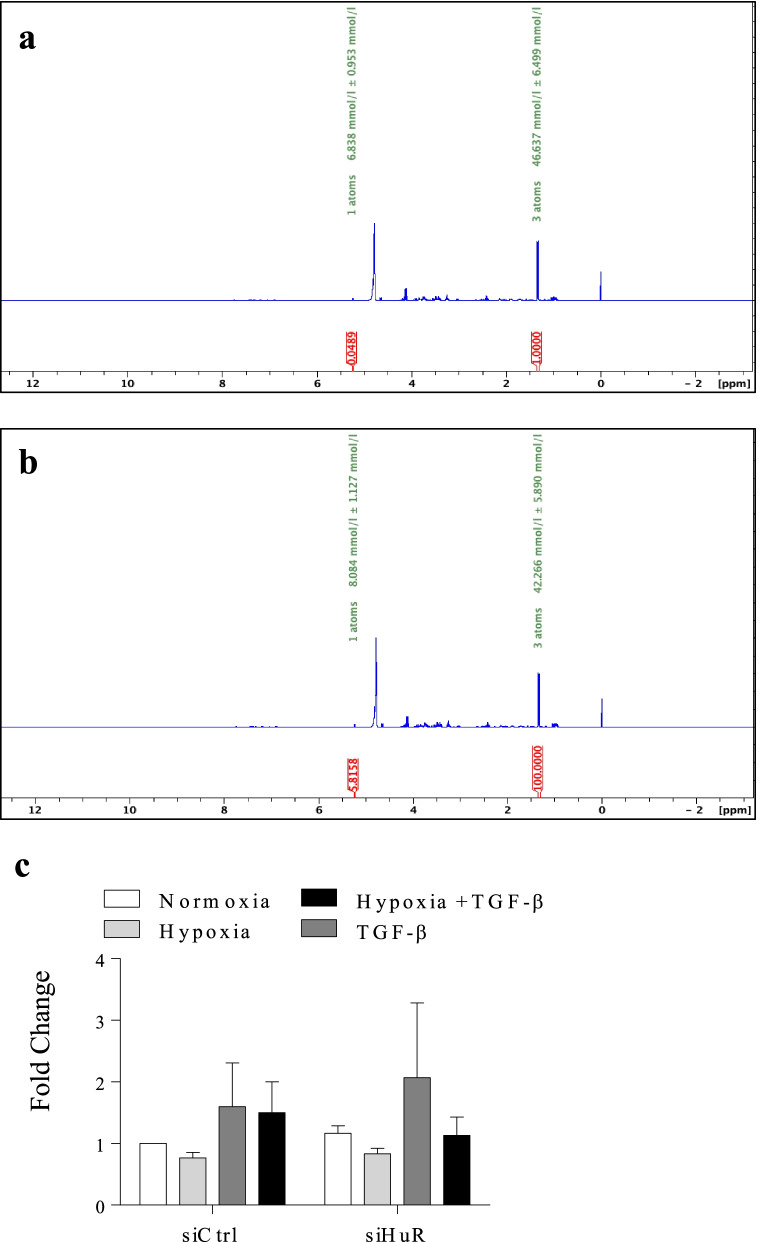
Lactate production does not change in response to HuR knock-down in HLFs. Sample spectra from HLFs transfected with siCtrl **a** and siHuR **b** that were treated with TGF-β1. Note the similar lactate and glucose peaks at 5.2 ppm and 1.3 ppm respectively **c** Quantification of lactate concentration relative to glucose between siCtrl- and siHuR-transfected HLFs. Values are represented as the mean ± SEM (n = 3 independent experiments using cells from 3 separate subjects)

**Fig. 10:**
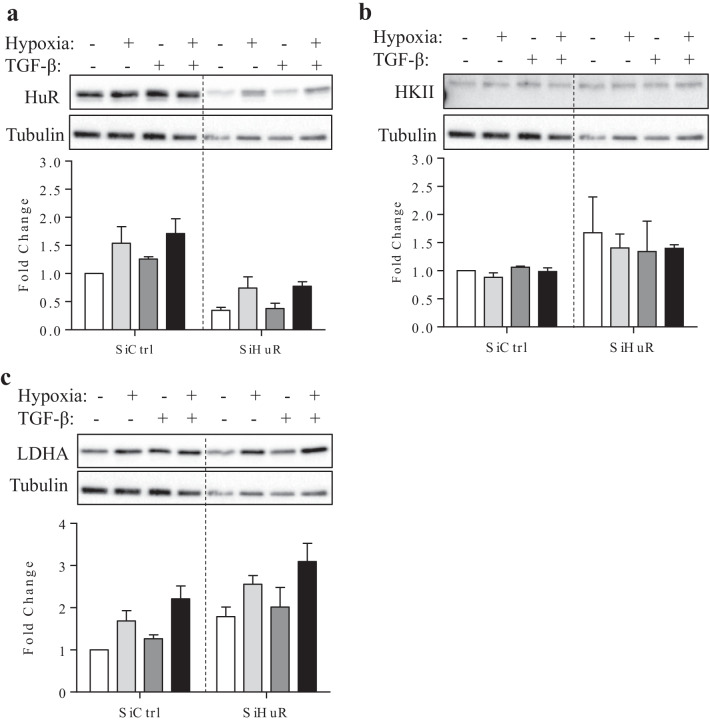
HuR knockdown does not affect select proteins of the glycolytic pathway. **a** HuR knockdown verification. **b** There is no effect of HuR knockdown on HKII protein levels. (**c**). HuR knockdown does not affect LDHA protein levels. Results are expressed as the mean ± SEM (n = 2 independent experiments)

We next investigated the role of HuR in the metabolic shift to glycolysis. After transfecting HLFs with HuR siRNA or control siRNA and treating with hypoxia, TGF-β1 and hypoxia with TGF-β1 for 72 h, we utilized ^1^H NMR to measure lactate production versus glucose consumption. Figure 9a and b represent sample spectra from TGF-β1-treated HLFs and demonstrate minimal changes in lactate production after HuR knock-down. Figure 9c shows that HuR does not play a significant role in lactate production under these experimental conditions. Finally, we evaluated whether HuR controls the expression of select markers of the glycolytic pathway including HKII and LDHA. Using siRNA to reduce HuR expression (Fig. 10a), these data show that HuR does not significantly control the protein expression of either HKII (Fig. 10b) or LDHA (Fig. 10c) under the exposure conditions used in this study. Thus, while silencing HuR expression via siRNA reduces TGF-β1 induction of fibroblast differentiation and ECM production, there is no significant change in glycolysis. Overall, our data show that HuR controls lung fibroblast differentiation to myofibroblasts and ECM production but does not play a significant role in metabolic reprogramming in HLFs.

## Discussion

IPF is a devastating and progressive disease that typically leads to respiratory failure, with a median survival of 3–5 years from diagnosis [2]. The exact cause of IPF remains unknown and the underlying mechanisms behind disease pathogenesis are poorly understood [30]. One of the main features of IPF is fibroblast-to-myofibroblast differentiation characterized by the expression of a-SMA and increased ECM production. An emerging feature of IPF is an increase in aerobic glycolysis and lactate production, resulting in pH-dependent activation of TGF-β1 and myofibroblast differentiation [10, 31, 32]. In this study, we focused on a novel link between myofibroblast differentiation, glycolysis and HuR. Current literature reveals that HuR regulates the stability and/or translation of target mRNA that encode proteins involved in pathogenic mechanisms that drive fibrotic disease. We recently reported that silencing HuR attenuates lung fibroblasts differentiation and collagen production [20]. We now sought to evaluate whether HuR regulates proteins of fibrotic and glycolytic pathways in lung fibroblasts upon treatment with TGF-β1 and hypoxia.

One of the more intriguing findings from this work was the combined effect of hypoxia and TGF-β1 on markers of myofibroblast differentiation and metabolic reprogramming. In this study, we hypothesized that HuR would mediate both fibroblast differentiation and metabolic reprogramming in response to hypoxia and TGF-β1 by stabilizing mRNAs of ECM and as well as those recognized as glycolysis markers. Our rationale was the fact that hypoxia-induced signaling underlies the pathogenesis of various life-threating lung diseases [33, 34]. In lung cancer, hypoxia is closely related to fundamental changes in tumor cells, resulting in resistance to cell death, increased angiogenesis and reprogramming energy metabolism including overexpression of the metabolic enzymes HKII and LDH, the net result of which increases glucose uptake and the switch to aerobic glycolysis [35]. With this rationale, the first step was to understand whether hypoxia affected differentiation and metabolic regulation in HLFs. We observed that hypoxia- when combined with TGF-β1- increased *ACTA2* mRNA compared to TGF-β1 alone. This effect was time-dependent, as the induction of *ACTA2* mRNA was seen only at 24 h; by 48 h post-treatment the effect of hypoxia in conjunction with TGFβ1 was not different from hypoxia alone. At the protein level, there was significantly less α-SMA and collagen expression from hypoxia together with TGF-β1. These data show that while hypoxia initially increases the transcriptional levels of *ACTA2*, there is reduced protein expression of α-SMA and collagens as well as lower lactate secretion. This was not due to changes *ACTA2* mRNA binding to HuR as evaluated by RIP-qPCR analysis. Alterations in translation control occur in the context of IPF [36] and one cellular pathway implicated in cellular protein synthesis is the PI3K/Akt/mTORC1 signal transduction pathway [37]; this pathway is also involved in metabolism [38]. Recent evidence points to an indirect role for HuR in regulating components of this pathway in hypoxic cells [39]. Although not evaluated in this study, it could be that HuR fine-tunes translation control via the PI3K/Akt/mTORC1, and thus account for the discrepancy between mRNA and protein expression caused by combined exposure to hypoxia and TGF-β. Based on our observation that there is less HuR translocation to the cytoplasm in response to hypoxia and TGF-β, this suggests that sequestration of HuR to the nucleus may contribute to the lower levels of α-SMA and collagen proteins that is observed with the co-treatment.

Concurrent with this, we also evaluated whether hypoxia would regulate metabolic reprograming. Upon exposure to only 1% O_2_ (thereby mimicking cellular hypoxic conditions), there was minimal induction of *LDHA* and *HKII* mRNA nor was there an increase in lactate production. This relative lack of induction was surprising, as we had hypothesized that extracellular lactate would increase in response to hypoxia alone. Moreover, previous work has shown that HIF-1α leads to increased LDH and myofibroblast differentiation [10]. In contrast, we observed that exposure to hypoxia reduced TGFβ1-induced lactate production and myofibroblast differentiation. Although previous reports show that hypoxia increases markers of myofibroblast differentiation and glycolysis [32, 40, 41], our results do not support that hypoxia is a factor that contributes to myofibroblast differentiation in the lungs. It should be noted that we did not evaluate other important metabolic proteins (e.g., PDH, PFK, GLUTs etc.) involved in metabolic reprogramming that may be altered in response to hypoxia. Discrepancies between our results and previous reports could be due to differences in experimental protocols (e.g., length of exposures) and cells involved (e.g., IPF-derived cells versus non-diseases cells used in this study; e.g., MRC-5 cells (a fetal fibroblast cell line) [31] and adventitial fibroblasts [41]. This is an important difference, as there is remarkable heterogeneity of fibroblast function between and within organs, including the lungs [42–44]. Moreover, studies showing fibroblast differentiation in response to hypoxia and TGF-β reported changes in mRNA levels (and not protein) [40], the results of which are in line with our own (e.g., hypoxia plus TGF-β increasing *ACTA* mRNA). Other studies relied on immunostaining to evaluate HIF-1α expression in IPF tissue and the bleomycin model [45], which does not show causation. However, based on our data, we conclude that hypoxia may actually slow down the effects of TGF-β1 (e.g., differentiation and metabolic reprogramming) by relegating HuR back to the nucleus.

The mechanism through which hypoxia reduces fibroblast differentiation, ECM levels and lactate production induced by TGF-β1 is not known, but there are several possibilities. First, this response may involve the RhoA GTPase signaling network. RhoA regulates the serum response factor (SRF) and myocardin related transcription factor (MRTF) signaling pathway, that is important for fibroblast-to-myofibroblast differentiation [46, 47]. Recent literature has demonstrated that TGF-β1 stimulates RhoA, allowing it to activate MRTF/SRF complex. Once the MRTF/SRF complex is activated, it induces the transcription of target genes, including α-SMA and collagen [48–50]. Interestingly, RhoA is inhibited by ARHGAP29, which is increased following hypoxia exposure [51, 52]. ARHGAP29 belongs to a class of regulatory proteins which leads to RhoA inhibition [52]. Furthermore, hypoxia induces the transcriptional up-regulation of adenyl cyclases that inhibit RhoA activity [53]. Another possibility- and the focus of this body of work- is understanding whether hypoxia controls the differentiation of lung fibroblasts via HuR. Experimental data has shown that HuR stabilizes mRNA primarily containing AREs by protecting mRNA from degradation machinery [54]. It should be noted that HuR also has the ability to decrease mRNA stability and/or translation in response to various stimuli [55–57] with HuR translocation to the cytoplasm leading to functional changes [58]. Our data show the localization of HuR in the cytoplasm is increased in response to both hypoxia and TGF-β1 alone. This agrees with a previous study whereby hypoxia increased cytoplasmic HuR [59]. HuR translocation to the cytoplasm suggests that it could be controlling the expression of ECM markers and lactate secretion induced by TGF-β1. Indeed, knock-down HuR in HLFs significantly attenuates TGF-β1 induction of α-SMA and collagen I protein, reenforcing the notion that HuR is a master regulator crucial for driving myofibroblast differentiation. Interestingly, knockdown of HuR increased HIF-1α protein except in response to hypoxia. suggesting a dynamic interplay between HuR and HIF-1α expression, an interplay that may be why there is less fibroblast differentiation in response to TGF-β despite similarities in HuR binding to target mRNA. However, silencing HuR did not alter levels of secreted lactate. Thus, our data suggest that HuR has a negligible role in regulating mRNA and/or enzymes involved in the glycolytic pathway. This differential role for HuR may be related to its ability under certain conditions (*i.e.,* hypoxia) to sequester mRNA in stress granules (SGs) [56, 60, 61]. SGs are ribonucleoprotein complexes that contain stalled mRNAs, pre-initiation factors and specific RNA binding proteins which allow cells to adapt and respond to stress [61, 62]. Studies have shown that hypoxia leads to the phosphorylation of eIF2α, resulting in the accumulation of the RNA pre-initiation complex 48S and promoting the formation of SGs [63, 64]. SGs contain HuR in addition to other RBPs that selectively bind to target mRNA with high ARE content and serve to keeping mRNA in a translationally-silent state. Formation of SGs has been associated with sequestering key components of pathways such as the NF-κB and p38/JNK pathways in response to hypoxia [65]. Thus, in response to hypoxic stress, HuR may sequester *ACTA2* and *Col1a1* mRNA in SGs, thereby reducing their translation and subsequent expression.

**Fig. 11:**
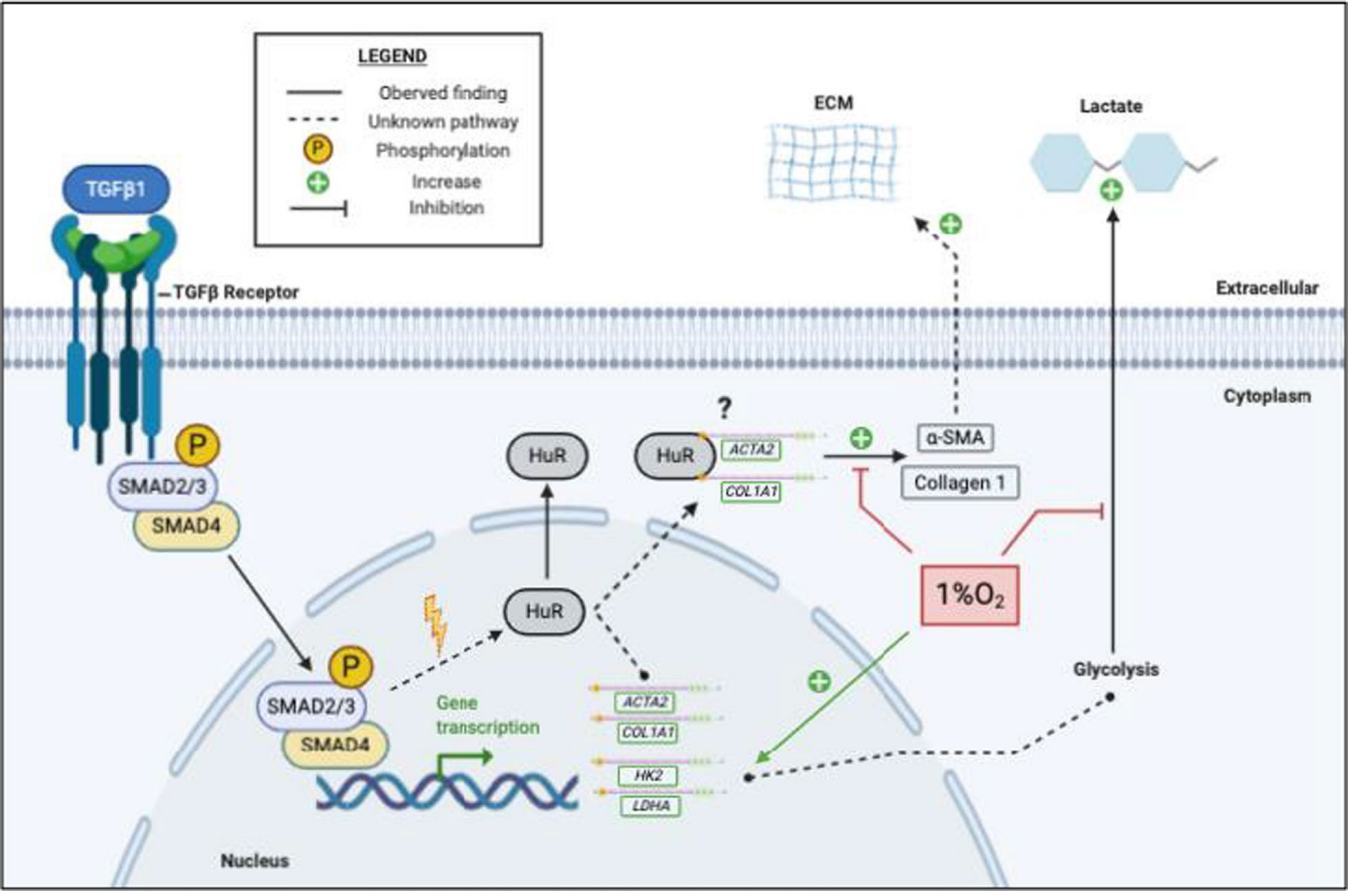
A schematic representation of the role of HuR in response to TGF-β1 and hypoxia. In response to TGF-β1 and 1% O_2_ (hypoxia), HuR translocates to the cytoplasm. HuR is crucial in the process of fibroblast differentiation and collagen production. Hypoxia greatly reduced TGF-β1-induced fibroblast differentiation and lactate production through an unknown mechanism

In conclusion, we are the first to report cytoplasmic translocation of HuR in human lung fibroblasts in response to hypoxia, TGF-β1 and hypoxia combined with TGF-β1. We also show that hypoxia unexpectedly abrogates TGF-β1-induced differentiation and slows metabolic reprogramming. Our work further elucidates the relationship between HuR and fibroblast differentiation using a model that exposes cells to TGF-β1 and hypoxia, a combination that replicates conditions seen in the more complex microenvironment of the lungs; Fig. 11 provides an overview of our findings, including that hypoxia reduces features of myofibroblast differentiation and glycolysis in response to TGF-β. Our study further supports the notion that HuR regulates TGF-β1-induced lung fibroblast differentiation but not glycolysis. Further research to investigate the relationship between HuR and fibrotic lung disease could lead to the development of novel therapies aimed at slowing down or even reversing the advancement of lung fibrosis.

## Conclusion

Our results show that HuR undergoes cytoplasmic shuttling in response to hypoxia in lung fibroblasts. HuR controls myofibroblast differentiation and ECM production but does not control metabolic reprogramming towards glycolysis. Further research is needed to understand how HuR controls pathogenic features associated with the devlopment of fibrotic lung disease.

## Data Availability

The data that support the findings of this study are available from the corresponding author upon reasonable request.

## Abbreviations

ACTA2: Actin alpha 2, smooth muscle gene
α-SMA: Alpha smooth muscle actin
ANOVA: Analysis of variance
ARE: AU rich element
COL1A1: Collagen type I alpha 1 chain gene
ECL: Enhanced chemiluminescence
ECM: Extracellular matrix
ELAVL1: Embryonic lethal abnormal vision-like protein 1
FBS: Fetal bovine serum
FN1: Fibronectin 1
HIF-1α: Hypoxia inducible factor 1 alpha
HKII: Hexokinase II
HLF: Human lung fibroblast
HuR: Human antigen R
HRP: Horseradish peroxidase
IF: Immunofluorescence
IP: Immunoprecipitation
ILD: Interstitial lung disease
IPF: Idiopathic pulmonary fibrosis
LDHA: Lactate dehydrogenase A
MRTF: Myocardin related transcription factor
^1^H NMR: Proton nuclear magnetic resonance
RIP-qPCR: RNA immunoprecipitation qPCR
qPCR: Quantitative polymerase chain reaction
RBP: RNA-binding protein
SEM: Standard error of the mean
SG: Stress granule
siRNA: Small-interfering RNA
SRF: Serum response factor
TGF-β1: Transforming growth factor beta 1
UTR: Untranslated region
